# „Die Familienanamnese bringt Licht ins Dunkel“

**DOI:** 10.1007/s00347-021-01401-9

**Published:** 2021-05-09

**Authors:** N. Woltsche, L. Posch-Pertl, S. Kamper, A. M. Haas, A. Wedrich

**Affiliations:** 1 Univ.-Augenklinik Graz, Medizinische Universität Graz, Auenbruggerplatz 4, 8036 Graz, Österreich; 2 Institut für Humangenetik, Medizinische Universität Graz, Graz, Österreich; 3 Abteilung für Augenheilkunde und Optometrie, Klinik Landstraße, Wiener Gesundheitsverbund, Wien, Österreich

## Falldarstellung

**Figure 1:**
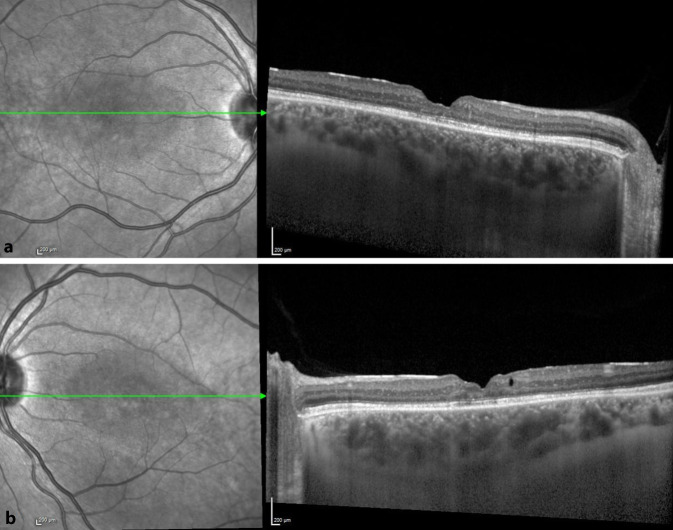

**Figure 2:**
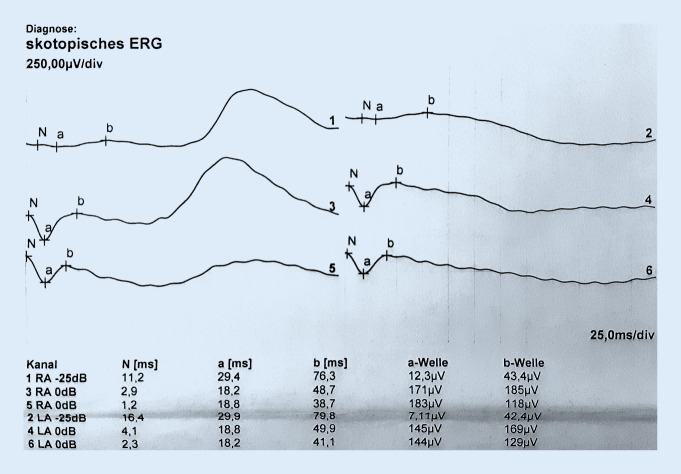

**Figure 3:**
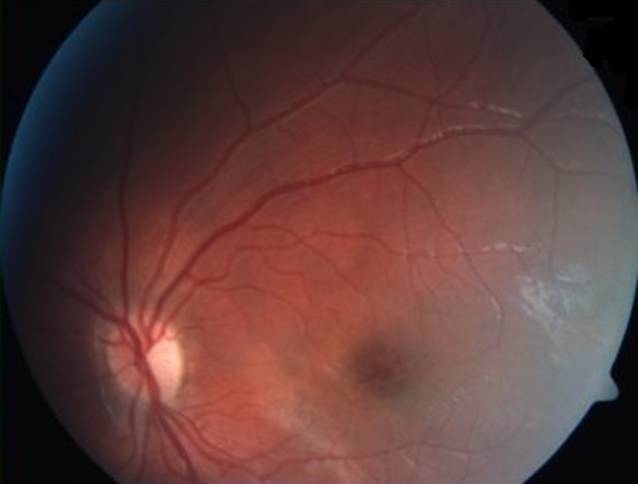

**Figure 4:**
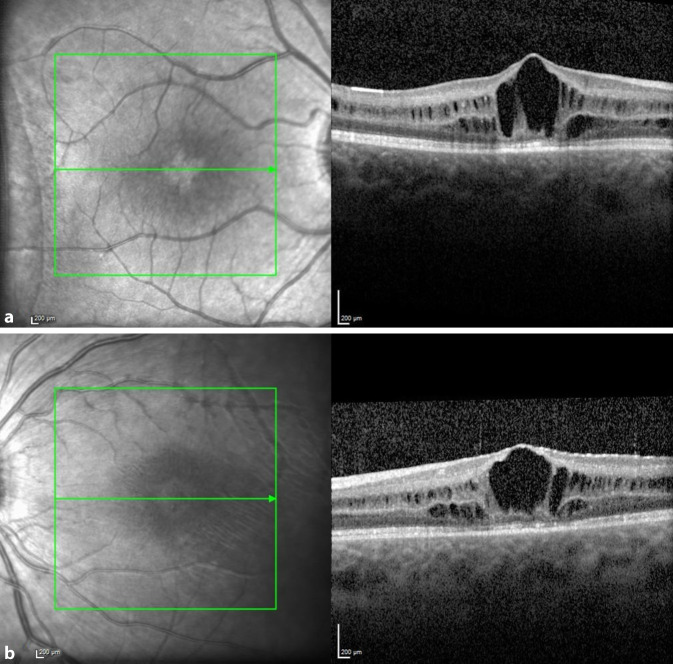

Im Februar 2018 wurde in der Netzhautsprechstunde der Univ.-Augenklinik Graz ein 38-jähriger Mann mit gleichbleibender Sehbeeinträchtigung seit dem Volksschulalter und Photophobie vorstellig. Bei augenärztlichen Untersuchungen 1999 und 2005 hatte er, wie auch sein Bruder, die Diagnose „Morbus Stargardt“ erhalten. Weder bei den Großeltern noch bei den Eltern oder weiteren Geschwistern bestanden okuläre Symptome oder Auffälligkeiten. Sein Bruder und er waren kinderlos. Eine genetische Abklärung sei bis dato nicht erfolgt, wurde aber jetzt vom Patienten gewünscht. Die ophthalmologische Untersuchung ergab einen unauffälligen äußeren Augenstatus, und der Fernvisus betrug sine correctione rechts 0,6 und links 0,4. In der optischen Kohärenztomographie (OCT) zeigten sich beidseits eine Foveaatrophie, eine irreguläre Konfiguration der fovealen Kontur und am linken Auge zusätzlich eine intraretinale Zyste (Abb. 1). Die Goldmann-Gesichtsfelduntersuchung war beidseits unauffällig, im multifokalen ERG (Elektroretinographie) zeigte sich jedoch beidseits eine Herabsetzung der Reizantworten, und auch im Ganzfeld-ERG war eine ausgeprägte skotopische Reduktion der b‑Welle bei normaler a‑Welle („elektronegatives ERG“, Abb. 2) auszumachen. Eine genetische Abklärung hinsichtlich „Zapfen-Stäbchen-Dystrophie“ blieb ergebnislos. Das OCT seines Bruders wies ähnliche Veränderungen mit einer Foveaatrophie und vereinzelten intraretinalen Zysten auf. Erst als der 8 Jahre alte Neffe der beiden Brüder (der Sohn ihrer Schwester) zufällig an unserer Klink vorstellig wurde, öffneten sich unsere Augen für die richtige Diagnose. Der Junge war seit seinem dritten Lebensjahr auffällig lichtempfindlich und hatte Sehprobleme in der Schule. Er präsentierte sich mit einem Fernvisus von 0,6 mit +2,0 dpt an beiden Augen und in der Fundoskopie zeigte sich eine Radspeichen- bzw. Sternfigur im zentralen Makulabereich (Abb. 3). Sein älterer Bruder zeigte keine okulären Auffälligkeiten. Zusammen mit der Familienanamnese und den OCT-Bildern brachte uns dieser Befund nun mit einem Schlag zur richtigen Diagnose der in dieser Familie vorliegenden Netzhautdystrophie (Abb. 4).

## Wie lautet Ihre Diagnose?

## Diagnose, Therapie, Verlauf

**Diagnose: **X‑linked juvenile Retinoschisis (XLRS)

Wie der Name schon sagt, handelt es sich um eine X‑chromosomal-rezessiv vererbte Erkrankung. Da Frauen über ein zweites, gesundes X‑Chromosom verfügen, erkranken sie meist nicht und sind Konduktorinnen. Die Wahrscheinlichkeit, dass Söhne von Konduktorinnen erkranken, beträgt 50 %. Die XLRS ist die häufigste Ursache einer makulären Dystrophie bei jungen Männern und weist eine Prävalenz von 1:5000 bis 1:20.000 auf. Bisher sind 191 verschiedene Mutationen im *RS1*-Gen bekannt, welche mit XLRS assoziiert sind. Die ursächliche *RS1*-Mutation konnte sowohl beim 38-jährigen Onkel als auch beim 8‑jährigen Neffen nachgewiesen werden. Das *RS1*-Gen kodiert für das Protein Retinoschisin, das von Fotorezeptoren und Bipolarzellen in den Interzellularraum sezerniert wird und für die interzelluläre Interaktion und Adhäsion der neurosensorischen Retina verantwortlich ist [1]. Die für die XLRS typischen Befunde in der ophthalmologischen Untersuchung sind eine Radspeichen- bzw. Sternfigur in der Fundoskopie, Schisisveränderungen in der inneren und zentral auch in der äußeren Körnerschicht im OCT sowie in vielen Fällen ein „elektronegatives ERG“ (b:a < 1,0). Häufig liegt auch eine Hypermetropie vor. Allerdings sind Abweichungen dieser typischen Charakteristika in der Literatur beschrieben. So zeigen bis zu 50 % der XLRS-PatientInnen kein elektronegatives ERG. Auch findet sich anstelle der typischen Aufspaltung der Netzhaut mit Radspeichenstruktur bei Patienten über 30 Jahre oft eine Atrophie der zentralen Netzhaut. Die Netzhautveränderungen sind höchstwahrscheinlich schon zum Zeitpunkt der Geburt vorhanden, jedoch werden die Kinder meist erst im Schulalter symptomatisch, typischerweise mit Leseschwierigkeiten und Photophobie. Zu den Komplikationen der XLRS zählen rhegmatogene Netzhautablösungen sowie Glaskörperblutungen aufgrund der potenziellen Traktionen und Schisisveränderungen auch in der Peripherie, welche wegen der Fragilität der Retina sehr schwer chirurgisch zu versorgen sind [2]. Es gibt jedoch bisher keine Anhaltspunkte, dass die foveale Netzhautdicke und Ausdehnung der zentralen zystischen Veränderungen mit dem Visus korrelieren [3].

In einzelnen Fallstudien konnte durch topische Gabe von Carboanhydrase-Inhibitoren eine Reduktion der intraretinalen Zysten gezeigt werden, weshalb wir beim 8‑jährigen Neffen eine Lokaltherapie mit 2‑mal täglich Dorzolamid 2 % Augentropfen einleiteten [4]. Eine signifikante Verminderung der intraretinalen Zysten wurde jedoch damit nicht erzielt. Eine neue therapeutische Option wird derzeit in einer monozentrischen Phase-I/IIa-Studie mit einem intravitreal verabreichten *RS1*-Adeno-assoziierten Virusvektor (AAV8-*RS1*) untersucht. Die gute Verträglichkeit und Sicherheit der Vektorverabreichung erlaubte eine Fortsetzung der Studie (NCT02317887). Interessanterweise könnte gerade bei der XLRS die intravitreale Applikation des Vektors gut funktionieren, da diese Netzhautdystrophie auch eine Alteration der internen Grenzmembran (ILM) aufweist. Dadurch wird eine Aufnahme des AAV-Vektors in die Netzhaut über die ILM ermöglicht, wie dies an *RS1*-Maus-Modellen gezeigt werden konnte [5].

## Fazit für die Praxis

Bei all den apparativen Möglichkeiten, über die wir heute verfügen, dürfen wir die Wichtigkeit einer exakten Anamnese – gerade wie in diesem Fallbeispiel, im Hinblick auf Vererbungsmuster bei hereditären Erkrankungen – nicht außer Acht lassen. Man sollte eine komplette Familienanamnese erheben und explizit nach Augenerkrankungen der (Ur‑)Großeltern (mütterlicher- und väterlicherseits), Eltern, Onkel/Tanten, Geschwister, Cousinen/Cousins, Kinder, Nichten/Neffen und Enkelkinder fragen. Die therapeutischen Optionen bei XLRS sind mit lokalen Carboanhydrase-Inhibitoren derzeit noch sehr beschränkt, jedoch laufen derzeit Studien zur Erforschung der Wirksamkeit von intravitrealer Gentherapie.
